# Nei-like DNA glycosylase 2 selectively antagonizes interferon-β expression upon respiratory syncytial virus infection

**DOI:** 10.1016/j.jbc.2023.105028

**Published:** 2023-07-08

**Authors:** Lang Pan, Yaoyao Xue, Ke Wang, Xu Zheng, Azharul Islam, Nisha Tapryal, Anirban Chakraborty, Attila Bacsi, Xueqing Ba, Tapas K. Hazra, Istvan Boldogh

**Affiliations:** 1 Department of Microbiology and Immunology, University of Texas Medical Branch at Galveston, Galveston, Texas, USA; 2 Department of Internal Medicine, University of Texas Medical Branch at Galveston, Galveston, Texas, USA; 3 Faculty of Medicine, Department of Immunology, University of Debrecen, Debrecen, Hungary; 4 Key Laboratory of Molecular Epigenetics of Ministry of Education, School of Life Science, Northeast Normal University, Changchun, Jilin, China

**Keywords:** oxidative stress, DNA damage, NF-κB, transcription regulation, inflammation, innate immunity, antiviral response

## Abstract

As part of the antiviral response, cells activate the expressions of type I interferons (IFNs) and proinflammatory mediators to control viral spreading. Viral infections can impact DNA integrity; however, how DNA damage repair coordinates antiviral response remains elusive. Here we report Nei-like DNA glycosylase 2 (NEIL2), a transcription-coupled DNA repair protein, actively recognizes the oxidative DNA substrates induced by respiratory syncytial virus (RSV) infection to set the threshold of IFN-β expression. Our results show that NEIL2 antagonizes nuclear factor κB (NF-κB) acting on the IFN-β promoter early after infection, thus limiting gene expression amplified by type I IFNs. Mice lacking *Neil2* are far more susceptible to RSV-induced illness with an exuberant expression of proinflammatory genes and tissue damage, and the administration of NEIL2 protein into the airway corrected these defects. These results suggest a safeguarding function of NEIL2 in controlling IFN-β levels against RSV infection. Due to the short- and long-term side effects of type I IFNs applied in antiviral therapy, NEIL2 may provide an alternative not only for ensuring genome fidelity but also for controlling immune responses.

Pulmonary infection by the respiratory syncytial virus (RSV) is a large burden on human health (1). Airway epithelial cells lining the respiratory tract render them primary targets for RSV replication, mounting the earliest elements of the host defense *via* the secretion of cytokines or chemokines (2). Among them, type I interferons (IFNs) are key cytokines that not only confer antiviral activities but also amplify immunomodulatory functions (3). Meanwhile, RSV infection can induce reactive oxygen species (ROS) that impact cellular defenses (4, 5). Redox imbalance by overproduction of ROS prone to impair DNA integrity, however, how DNA damage induced by virus infection plays an active role in IFN regulation is incompletely defined.

The most abundant DNA lesions are oxidatively modified bases, which are primarily repaired *via* the base excision repair (BER) pathway (6). DNA glycosylases specifically recognize the modified bases, removing them by hydrolyzing the glycosidic bond and leaving behind an apurinic/apyrimidinic (AP) site, or cleaving the sugar-phosphate chain with an accompanied AP-lyase activity (7). Multiple glycosylases have evolved with overlapping substrate specificity and repair processes, which is thought to be the reason why single knock-out models of individual BER enzymes show no serious phenotype. However, mouse models of single Nei-like DNA glycosylases (NEIL) 1 and NEIL2, NEIL1/NEIL2 double, and NEIL1/NEIL2/NEIL3 triple knock-out result in metabolic stress and susceptibility to inflammation but no significant mutational load (8), which has strengthened the hypothesis that the NEIL enzymes are not simply back-up enzymes for each other but enzymes that have distinct roles *in vivo* beyond canonical repair. Moreover, NEIL2 knockout (*Neil2*^−/−^) mice are hyper-responsive to inflammatory agents (9, 10), which is in sharp contrast to 8-oxoguanine DNA glycosylase (OGG1) knockout (*Ogg1*^−/−^) mice that display resistance to inflammatory and allergic stimuli (11, 12). The best-established function for NEIL2 is excising oxidatively modified cytosine product from DNA bubble (13), which represents transient intermediates that preferentially formed during active transcription (14). Taken together, these reports point to a NEIL2 function in the processing of gene expression that is involved in the innate immune response.

Here, we demonstrate the basis of NEIL2 in the regulation of IFN-β levels upon RSV infection. NEIL2 specifically recognizes the oxidatively modified DNA in RSV-infected cells, including the gene loci of the IFN-β promoter. NEIL2 antagonizes NF-κB enrichment on chromatinized DNA that safeguards against exuberant IFN-β expression and proinflammatory genes. *Neil2*^−/−^ mice show high sensitivity to RSV infection with increased weight loss and tissue injury in the lung. The addition of NEIL2 protein sets the threshold of IFN-β levels, thus ameliorating immune pathogenesis. Altogether, our studies shed light upon the mechanism of RSV-induced immunopathology can be limited by DNA glycosylase NEIL2.

## Results

### NEIL2 recruited to the oxidative DNA substrates in RSV-infected cells

**Figure 1:**
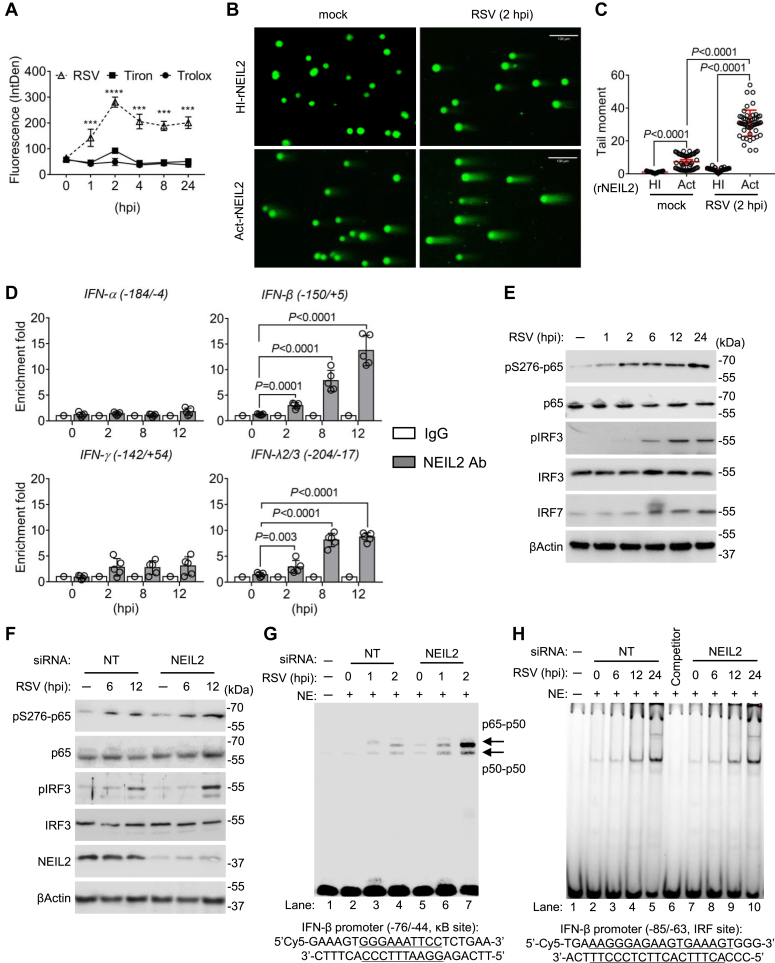
**ROS-induced recruitment of activated proteins to IFN-β promoter upon RSV infection.***A*, ROS assay after RSV infection with or without superoxide scavenger treatment. hSAECs were pre-incubated with Tiron (10 mM) or Trolox (200 mM) for 15 min, followed by RSV infection (MOI = 1) for the indicated time. ROS were detected *via* CellROX Green Reagent by measuring fluorescence intensity. Data are presented as mean ± SD from three biological replicates, two-way ANOVA. ∗∗∗*p* < 0.001. *B*, representative images of FLARE coupled comet assays from mock or RSV infected (2 h p.i.) hSAECs. Scale bar, 130 μm. *C*, quantification of comet tail moment shown in *B* (n = 50 from three biological replicates). *D*, enrichment of NEIL2 to the IFNs promoter in hSAECs at indicated hours p.i. ChIPs were performed using antibody against NEIL2, and IgG serves as negative control. n = 5 from three biological replicates. *C* and *D*, data are expressed as mean ± SD, two-tailed, unpaired Student’s *t*-tests. *E*, hSAECs were infected by RSV for indicated time, whole cell lysates were prepared and subjected to western blotting with indicated antibodies. *F*, hSAECs were transfected with non-targeted (NT) and NEIL2 targeted siRNA for 24 h, followed by RSV infection for indicated time. Whole cell lysates were prepared and subjected to blotting with indicated antibodies. *G*, nuclear extracts were subjected to EMSA with oligo containing κB site. *H*, nuclear extracts were subjected to EMSA with oligo-containing IRF site. *E*–*H*, representative images are shown from three biological replicates.

ROS have been established as important signaling messengers in response to RSV infection (4, 15). We then established the kinetic changes in RSV-induced ROS levels in human small airway epithelial cells (hSAECs) using a fluorogenic probe. Results show that RSV-induced oxidative burst peaked at 2 h post infection (p.i.), and the treatment with ROS scavengers, Tiron (a global O_2_^−^ scavenger) and Trolox (globally scavenges OOH and OOR) (16), inhibited the fluorescent confirming that the signal was due to ROS generation (Fig. 1*A*). To directly monitor the impact of RSV infection on host DNA integrity, we performed Fragment Length Analysis using Repair Enzyme(s) (FLARE) coupled comet assays. Active (Act) recombinant (r)NEIL2 digestion was used to detect oxidative DNA base damage caused by RSV infection, and the heat-inactivated (HI) rNEIL2 was used as negative control. We observed that significant global-DNA damage increased at 2 h p.i. with Act-rNEIL2 digestion (Fig. 1*B*), as measured by comet tail moment (Fig. 1*C*). This result suggests that RSV infection causes oxidative DNA base lesions, and the damaged DNA substrates can be specifically recognized by NEIL2. We next tested whether the DNA damage recognized by NEIL2 localizes in the promoters of IFNs genes. Chromatin immunoprecipitation (ChIP) analysis using NEIL2 antibody shows 5 to 15-folds enrichment on IFN-β and IFN-λ2/3 promoters within 2∼12 h p.i but no significant enrichment on corresponding regions of IFN-α and IFN-γ promoters (Fig. 1*D*). Taken together, these results suggest that RSV infection activates NEIL2 to locate damaged sites including IFN-β promoter.

### NEIL2 antagonizes IFN-β expression by suppressing DNA occupancy of NF-κB

RSV infection activates transcription factors including NF-κB (17), we then tested the activation of *trans* factors, NF-κB/RelA and IRF3, through phosphorylation. RSV-infected hSAECs showed detectable phosphorylation of NF-κB/RelA subunit (pS276-p65) at 1 to 2 h p.i, whereas the phosphorylation of IRF3 started to show from 6 to 12 h p.i (Fig. 1*E*). The protein levels of NF-κB/RelA and IRF3 are stable within 24 h infection, while the level of IRF7 is increased at 12 h p.i (Fig. 1*E*). These results are consistent with the previous report that NF-κB activation is responsible for the early induction of IFN-β expression and a secondary phase characterized by high levels of IRF7 expression (18). Neither the protein levels of NF-κB/RelA and IRF3 nor the phosphorylation was affected by siRNA-mediated NEIL2 knockdown within 12 h p.i examined (Fig. 1*F*), indicating that NEIL2 has no effect on the protein and phosphorylated levels of NF-κB/RelA and IRF3. After confirming the activation of NF-κB and IRF3 upon RSV infection, we performed EMSA analysis using Cyanine-5 (Cy5)-labeled κB site (GGGAAATTCC) and IRF site (AAGGGAGAAGTGAAAGT) containing oligo nucleotides. In comparison to mock, we observed increased DNA-protein complex in the presence of nuclear extracts (NEs) from RSV infected cells (Fig. 1*G*, lane 3–4, and Fig. 1*H*, lane 3–5), while siRNA-mediated NEIL2 knock down shows stronger shift with κB site oligo, but not IRF3 (Fig. 1*G*, lane 6–7 and Fig. 1*H*, lane 8–10). These results suggest that NEIL2 is interfering with the DNA binding of NF-κB.

**Figure 2:**
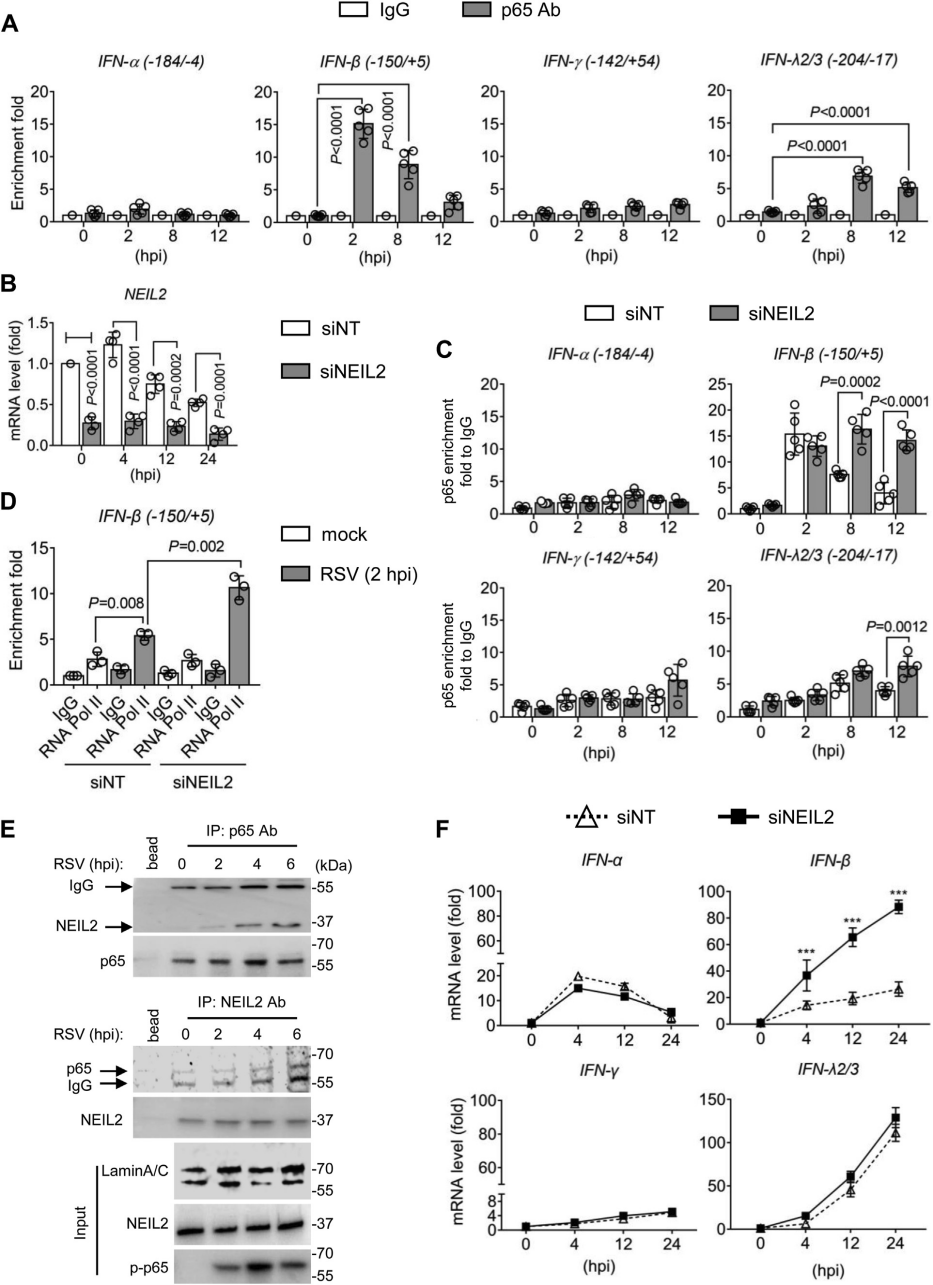
**NEIL2 actively suppresses IFN-β expression in RSV-infected hSAECs.***A*, enrichment of NF-κB/RelA to IFNs promoter at indicated hours p.i (MOI = 1). IgG serves as a negative control. n = 5 from three biological replicates. *B*, hSAECs were transfected with non-targeted (NT) and NEIL2 targeted siRNA for 24 h, followed by RSV infection for indicated time. NEIL2 mRNA level was measured by qRT-PCR. n = 4 from three biological replicates. *C*, chromatin was immunoprecipitated by antibody against NF-κB/RelA, and eluted DNA was subjected to qRT-PCR with primers amplifying promoter regions of IFNs. n = 5 from three biological replicates. *D*, chromatin was immunoprecipitated by antibody against RNA Pol II, and eluted DNA was subjected to qRT-PCR with primers amplifying IFN-β promoter. n = 3 from three biological replicates. *A*–*D*, data are expressed as mean ± SD, two-tailed, unpaired Student’s *t*-tests. *E*, co-IP analysis using nuclear proteins. Immunoprecipitation was performed using anti-NF-κB/RelA, and immunoblotted with anti-NEIL2, or verse versa. Protein A/G Mix Magnetic Beads adding to the lysates without antibody serves as negative control. LaminA/C represents input from nuclear protein. Representative images are shown from three biological replicates. *F*, total RNA was extracted from hSAECs, and mRNA level was measured by qRT-PCR. Data are expressed as mean ± SD from three biological replicates. *p* values were calculated by two-way ANOVA, ∗∗∗*p* < 0.001.

We then examined the enrichment of NF-κB/RelA in the same region as NEIL2 enriched on the IFNs promoter. ChIP experiments show that NF-κB/RelA binding to IFN-β promoter peaked as early as 2 h p.i, and gradually decreases by at 12 h p.i, and on IFN-λ2/3 promoter that peaks at 8 h p.i (Fig. 2*A*). There is no significant NF-κB/RelA enrichment on IFN-α and IFN-γ promoter. These results are consistent with previous reports that the regions we amplified within IFN-β and IFN-λ2/3 promoter contain κB sites but not IFN-α and IFN-γ promoter (19). We then depleted NEIL2 (Fig. 2*B*) and tested NF-κB/RelA and active RNA Pol II enrichment to the promoter of IFN-β. Compared with non-targeting (NT) siRNA, NEIL2-targeted siRNA results in enhanced DNA occupancy of NF-κB/RelA and active RNA Pol II to IFN-β promoter immediately at 2 h p.i (Fig. 2, *C* and *D*), and some level of NF-κB/RelA enrichment to IFN-λ2/3 promoter from 8 h p.i (Fig. 2*C*). These results indicate NEIL2 antagonizes NF-κB acting with the IFN-β promoter, thus limiting active RNA Pol II enrichment in the initiation of transcription upon RSV infection. Indeed, in the presence of BMS 345541, a highly selective inhibitor of I kappa B kinase, RSV-induced expressions of IFN-β and pro-inflammatory mediators, like TNFα, CCL20, CXCL10, and IL6, are significantly decreased in hSAECs (Fig. S1), suggesting the dictator role of NF-κB in RSV-induced gene expression. Previously, we showed that NEIL2 was displaced by activated NF-κB from the promoters through interacting with regulatory subunit of NF-κB/RelA (10). We then performed Co-IP experiments using NEs. Results show that NEIL2 and NF-κB/RelA are in the same immune-complex after RSV infection (Fig. 2*E*), indicating that in RSV-infected cells, NEIL2-mediated decrease in the NF-κB enrichment to promoter is due to protein–protein interaction.

**Figure 3:**
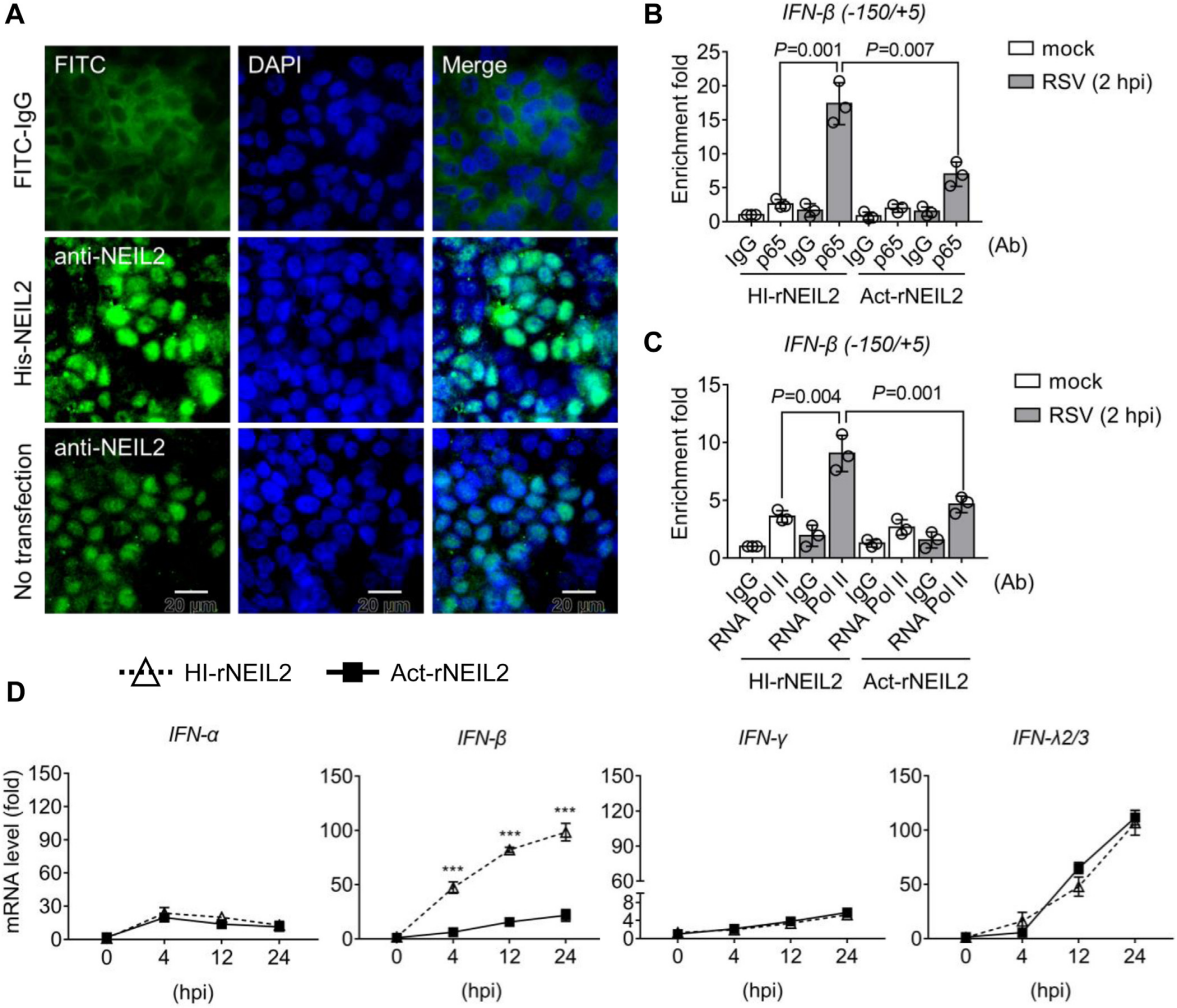
**The effect of recombinant NEIL2 on RSV-induced IFN-β expression.***A*, representative IF images of rNEIL2 transfection after 24 h in hSAECs. FITC-IgG serves as transfection control. Scale bar, 20 μm. *B*–*D*, hSAECs were transfected by NEIL2 targeted siRNA for 24 h, followed by supplementation of heat-inactivated (HI) and active (Act) rNEIL2. Chromatin was prepared for ChIP experiments after RSV infection for 2 h with the antibody against NF-κB/RelA (*B*) and RNA Pol II (*C*) to the IFN-β promoter. Data are expressed as mean ± SD, n = 3 from three biological replicates, two-tailed, unpaired Student’s *t*-tests. *D*, mRNA level was measured by qRT-PCR. Data is expressed as mean ± SD from three biological replicates. *p* values were calculated by two-way ANOVA, ∗∗∗*p* < 0.001.

After exploring the mechanism of NEIL2 in regulating *trans* effects, we next assessed the involvement of NEIL2 in gene expression by siRNA-mediated gene depletion. In NEIL2-depleted cells, the mRNA levels of IFN-β increased significantly at 4 h p.i, while the expression of IFN-α, IFN-γ, and IFN-λ2/3 showed no change in comparison to non-targeted siRNA transfected cells (Fig. 2*F*). We note that IFN-α expressed as early as 4 h p.i., IFN-γ expression level is low in hSAECs and the expression of IFN-λ2/3 is accumulating at 24 h p.i. Given that NEIL2 is a DNA repair protein, we wondered whether the IFN-β expression can be antagonized by other DNA repair proteins. We then tested the effects of OGG1 and 7,8-dihydro-8-oxoguanine triphosphatase (MTH1) in RSV-induced gene expression. Results show that siRNA-targeted OGG1 decreases the expression of IFN-α, IFN-β, TNFα, CCL20, CXCL10, and IL6, while no effect on IFN-γ in hSAECs (Fig. S2). There is no significant of MTH1 targeted siRNA in gene expression. These results are consistent with previous report that OGG1 facilitates innate immune response upon RSV infection (20). To test whether IFNs gene expression in NEIL2-depleted cells can be reversed after rNEIL2 addition, we supplemented rNEIL2 following siRNA-targeted NEIL2 depletion in hSAECs (Fig. 3*A*). ChIP assays show the enrichment of NF-κB/RelA and active RNA Pol II to the IFN-β promoter are reduced with rNEIL2 addition (Fig. 3, *B* and *C*), and the mRNA level of IFN-β is suppressed by rNEIL2 (Fig. 3*D*). These results indicate the reduced expression of IFN-β is due to the reduced recruitment of p65 and RNA Pol II on the promoter.

### Increased IFN-β expression in *Neil2*^−/−^ mice

**Figure 4:**
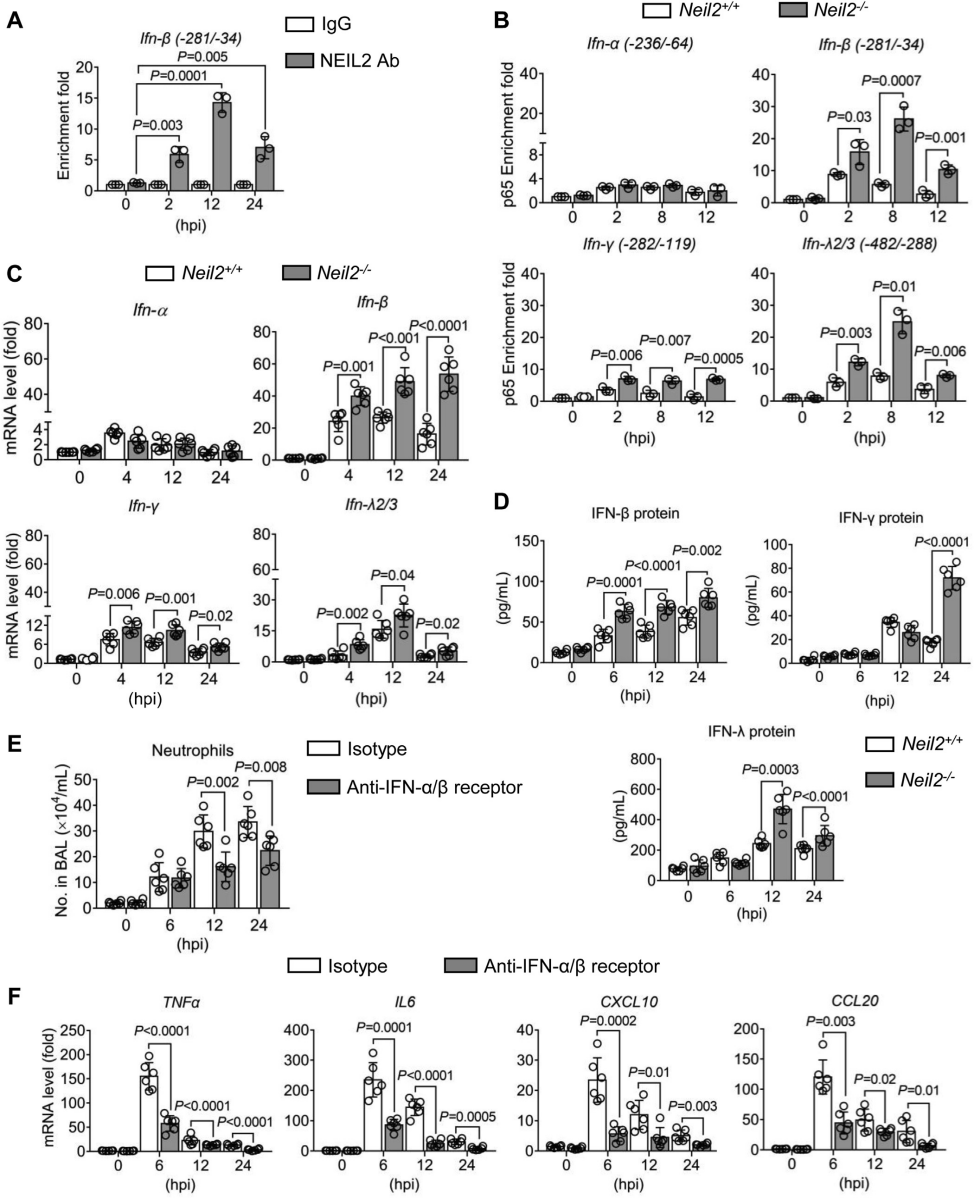
**NEIL2 controls IFNs expression in the lungs.** Mice were intranasally infected with 10^6^ PFU of RSV for the indicated time. *A*, chromatin from the lungs of *Neil2*^+/+^ mice was immunoprecipitated with an antibody against NEIL2. DNA was extracted and amplified with primers corresponding to the IFN-β promoter. n = 3 mice from three biological replicates. *B*, chromatin from the lungs of *Neil2*^+/+^ and *Neil2*^−/−^ mice was immunoprecipitated with antibodies against NF-κB/RelA. DNA was extracted and amplified with primers corresponding to IFN promoter regions. n = 3 mice from three biological replicates. *C*, mRNA levels of IFNs in the lungs were measured by qRT-PCR at indicated time p.i. n = 6 mice from three biological replicates. *D*, the concentrations of IFN-β, IFN-γ, and IFN-λ protein in the BALF were measured by ELISA at indicated hours p.i. n = 6 mice from three biological replicates. *E* and *F*, *Neil2*^+/+^ mice were treated with the antibody against IFN-α/β receptor for 24 h prior to infection with RSV (10^6^ PFU) for indicated time. Isotype antibody (IgG) serves as control. *E*, number of neutrophils infiltrated in the BALF. n = 6 mice from three biological replicates. *F*, RNA was isolated from lungs, and gene expression levels of TNFα, IL-6, CXCL10 and CCL20 were determined by qRT-PCR. n = 6 mice from three biological replicates. Data are expressed as mean ± SD, two-tailed, unpaired Student’s *t*-tests.

Our NEIL2 overexpression experiments showed the antagonization of IFN-β expression, which we wanted to validate *in vivo*. To this end, we first examined the recruitment of NEIL2 to the IFN-β promoter in the lungs of *Neil2*^+/+^ mice after RSV infection. Consistent with the results shown in cell culture, the peak of NEIL2 occupancy was detected at 12 h p.i and then decreased at 24 h p.i but still significantly associated with the promoter (Fig. 4*A*). We next utilized *Neil2*^−/−^ mice (Fig. S3) to test NF-κB/RelA recruitment. In *Neil2*^+/+^ mice, we observed the recruitment of NF-κB/RelA to *Ifn-β* and *Ifn-λ2/3* promoters as early as 2 h p.i. and decreased at 12 h p.i (Fig. 4*B*), indicating the early NF-κB activation in the lung. In the absence of NEIL2, NF-κB/RelA recruitment to the *Ifn-β*, *Ifn-γ* and *Ifn-λ2/3* promoters are even higher and sustained to 12 h p.i (Fig. 4*B*). Because the selective assembly of NF-κB to the promoter is an early event and highly dynamic, these results demonstrate that NEIL2 recruitment to the damaged sites in the promoter selectively antagonizes the DNA binding of NF-κB/RelA through protein-protein interactions. Compared with *Neil2*^+/+^ mice, the mRNA levels of *Ifn-β*, *Ifn-γ*, and *Ifn-λ2/3* are increased as early as 4 h p.i. in *Neil2*^−/−^ mice (Fig. 4*C*). Moreover, in the absence of NEIL2 by siRNA-mediated depletion in hSAECs and *Neil2*^−/−^ mice, the expression of IFN-β is 1.5 to 2.5 folds higher than the presence of NEIL2 under mock-infection (Fig. S4). This result suggests a role of NEIL2 in regulating IFN-β expression under both basal and induced conditions. The protein levels of IFNs in the bronchoalveolar lavage fluid (BALF) were measured by ELISA. In *Neil2*^−/−^ mice, the levels of IFN-β, IFN-λ, and IFN-γ are significantly higher at 12 to 24 h p.i. compared to *Neil2*^+/+^ mice (Fig. 4*D*). These results suggest that NEIL2 can limit IFN-β expression in the murine model.

### Suppression of IFN-β levels by NEIL2 ameliorates immunopathology upon RSV infection

Type I IFNs strongly amplify the inflammatory response, including higher expression of proinflammatory cytokines and chemokines (5, 21). To evaluate the contribution of IFN-β in inflammation, we first delivered IFN-α/β receptor antibody to the C57BL/6 mice by the intranasal route and inoculated with RSV. At 12 to 24 h p.i, we observed a significant reduction in the numbers of neutrophils infiltrated in the BALF of mice pre-treated with IFN-α/β receptor neutralizing antibody (Fig. 4*E*). The expression levels of TNFα, CCL20, CXCL10, and IL6 are significantly decreased at 6 to 24 h p.i. in the lung with neutralizing antibody (Fig. 4*F*). These results suggest that in the absence of NEIL2, uncontrolled type I IFNs levels may amply pro-inflammatory gene expression.

**Figure 5:**
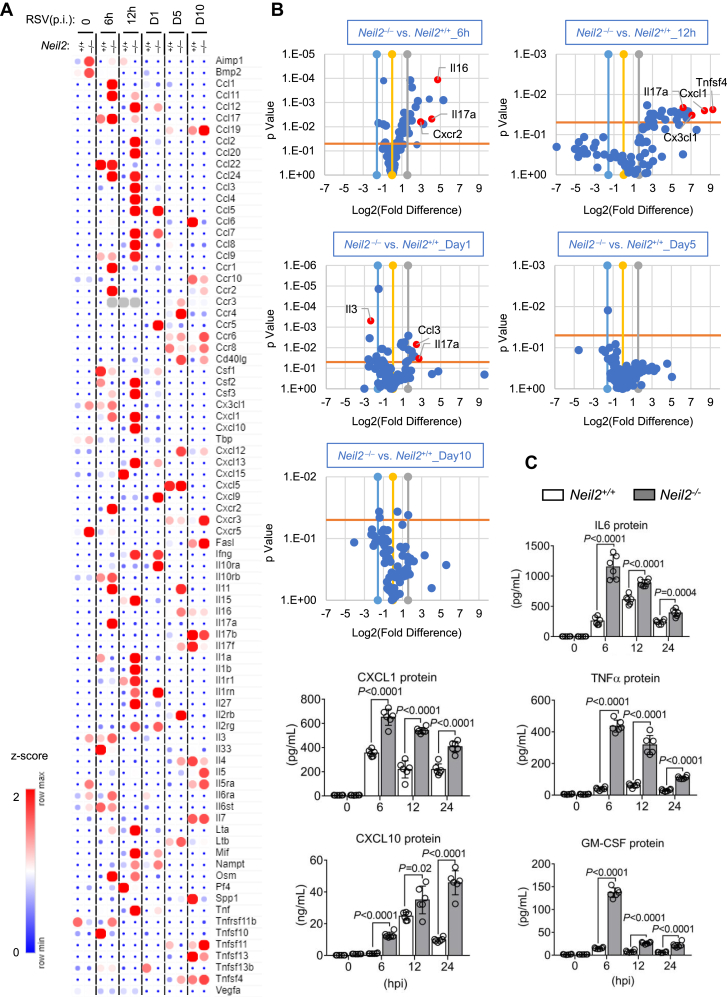
**Expression of inflammatory mediators in *Neil2***^**+/+**^**and *Neil2***^**−/−**^**mice after RSV infection.** Mice were RSV infected *via* intranasal route (10^6^ PFU per mouse). *A*, hierarchical clustering of inflammatory chemokine/cytokines. RNA was isolated from lungs and measured by RT^2^ Profiler PCR Array at indicated time p.i. The color bar indicates the z-score. *B*, volcano plots show pro-inflammatory gene transcripts enriched in the lungs at indicated time p.i. *Blue* and *gray lines* set a threshold for fold change (Log2), and the *orange line* set threshold for the *p* value. *C*, the concentrations of inflammatory cytokine and chemokine in BALF measured by Bio-Plex Chemokine analysis at the indicated time. Data are expressed as mean ± SD, n = 5 from three biological replicates, two-tailed, unpaired Student’s *t*-tests.

To extend the above results, we evaluated inflammatory gene expression profiles in *Neil2*^+/+^ and *Neil2*^−/−^ mice to gain further insight into the role of NEIL2 in response to RSV infection. Compared with *Neil2*^+/+^ mice, the mRNA levels of secreted proteins are significantly higher in *Neil2*^−/−^ mice at 6 to 12 h p.i, such as TNF, CCL2, CCL3, CCL4, CXCL1, and CXCL10 (Fig. 5, *A* and *B*). We extended the sampling period to 10 days p.i, the peak in fold difference of gene expression in *Neil2*^−/−^ *versus Neil2*^+/+^ mice lung is at 6 to 12 h p.i, during which time the innate antiviral responses are triggered. However, at later time points (day 5∼10 p.i.), there was no significant difference at gene expression in the lungs of *Neil2*^−/−^ *versus Neil2*^+/+^ mice (Fig. 5*B*). Concentrations of proinflammatory cytokines and chemokines were additionally measured in BALF using a multiplex approach. For some cytokines (IL-6, CXCL1, TNFα, CXCL10 and GM-CSF), there are 2 to 10 times higher levels in *Neil2*^−/−^ mice compared to *Neil2*^+/+^ at 6 to 24 h p.i (Fig. 5*C*). These results indicate that the role of NEIL2 is significant in the early response of innate antiviral responses.

**Figure 6:**
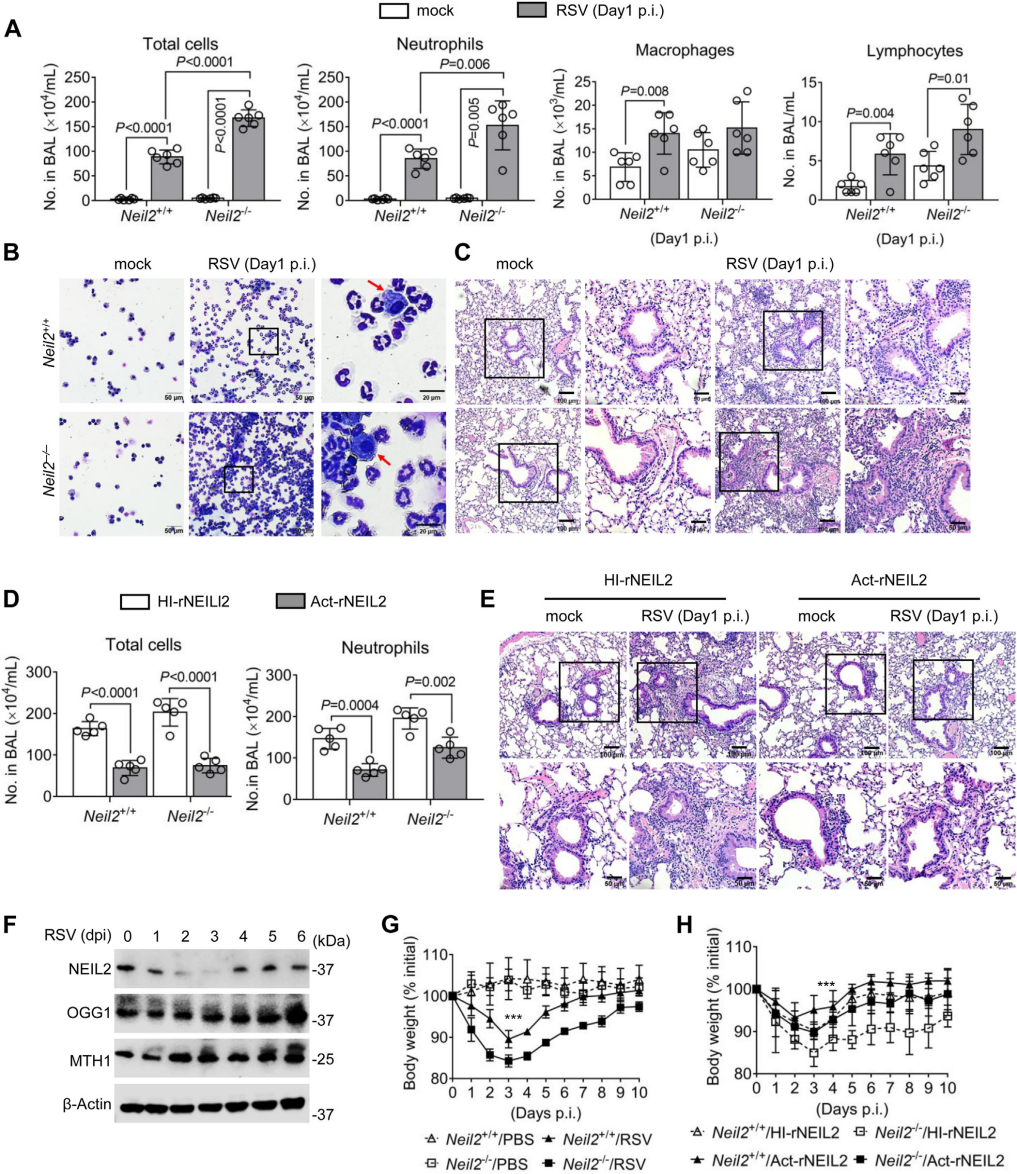
**NEIL2 ensures optimal host fitness without collateral tissue injury.***Neil2*^+/+^ and *Neil2*^−/−^ mice were intranasally infected with RSV (10^6^ PFU per mouse) for the indicated time. *A*, total cell and differential cell counts in the BALF are expressed as the number of cells per milliliter. Data are mean ± SD, n = 6 from three biological replicates, two-sided, unpaired Student’s *t* test. *B*, representative images of infiltrate cells in BALF. *Arrows* indicate macrophages. The scale bar represents 50 μm in lower magnification, and 20 μm with zoom in. *C*, representative H&E staining of lung sections. The scale bar represents 100 μm in lower magnification, and 50 μm with zoom in. *D*, total cellular-infiltrates, and neutrophil numbers in BALF at day 1 p.i. *Neil2*^+/+^ and *Neil2*^−/−^ mice were transfected with heat-inactivated (HI) or activate (Act) rNEIL2 prior to RSV-infection. Data are mean ± SD, n = 5 from three biological replicates, two-sided, unpaired Student’s *t* test. *E*, representative H&E staining of lung sections from *Neil2*^−/−^ mice transfected with heat-inactivated (HI) and activate (Act) rNEIL2. The scale bar represents 100 μm in lower magnification, and 50 μm with zoom in. *F*, Western blot shows the protein levels of NEIL2, OGG1 and MTH1 in the lungs of *Neil2*^+/+^ mice at indicated days p.i (dpi). Blot is from six mice per group pooled from three biological replicates. *G*, body weight in *Neil2*^+/+^ and *Neil2*^−/−^ mice at indicated days p.i. *H*, body weight in *Neil2*^+/+^ and *Neil2*^−/−^ mice supplemented heat-inactivated (HI) and activate (Act) rNEIL2 at indicated days p.i. *G* and *H*, data are mean ± SD, n = 6 from three biological replicates, two-way ANOVA, ∗∗∗*p* < 0.001.

At 24 h p.i., neutrophils significantly increased in the infiltrates of airway lumen (Fig. 6, *A* and *B*). *Neil2*^−/−^ mice exhibit higher percentage of neutrophils in the BALF at day 1 to 3 p.i. compared to *Neil2*^+/+^ mice (Fig. S5). Consistently, aggravated immunopathology was observed in *Neil2*^−/−^ mice, with increased presence of inflammatory cell infiltrates in peri-bronchial and parenchymal areas of the lung at day 1 p.i (Fig. 6*C*). Notably, this was associated with heightened production of IFN-β that triggers immunopathology in *Neil2*^−/−^ mice. Taking that the role of rNEIL2 in decreasing IFN-β levels in hSAECs, we tested further the impact of rNEIL2 in the protection against RSV-induced immunopathology by supplement rNEIL2 in mice (Fig. S6). Treatment with Act-rNEIL2 significantly lowered the total cell infiltrated into the airway lumen (Fig. 6*D*) among which neutrophil numbers significantly decreased (Figs. 6*D* and S7). The tissue injury also ameliorated by Act-rNEIL2 (Fig. 6*E*). Next, we wondered how NEIL2 expressed in the wildtype mice upon RSV infection as well as other BER proteins in the lungs of infected C57BL/6 mice. Unexpectedly, RSV infection significantly decreased the expression of endogenous NEIL2 at both transcript and protein levels, but not OGG1 or MTH1, at day 1 to 3 p.i (Figs. 6*F* and S8). These results suggest that NEIL2 downregulation ensures the effective antiviral response possibly through increasing IFN-β expression. At day 3 p.i, weight loss in *Neil2*^+/+^ mice is limited to only 10% of the total weight, while in *Neil2*^−/−^ mice reaches to >15% (Fig. 6*G*). *Neil2*^−/−^ and *Neil2*^+/+^ mice that were supplemented with Act-rNEIL2 experienced lower body weight loss relative to HI-rNEIL2 (Fig. 6*H*). These results suggest a role of NEIL2 in ameliorating tissue injury through suppression of IFN-β without compromising host fitness upon RSV infection.

## Discussion

This study uncovered a previously unknown function of NEIL2 in actively responding to RSV infection and specifically regulating IFN-β expression. ChIP and EMSA experiments demonstrate the antagonism of NEIL2 with NF-κB acting on the *cis*-regulatory regions of IFN-β and subsequently reflected in the alteration of biological outputs, such as transcriptional activity. Through genetic and biochemical modulation, our data demonstrate that NEIL2 is necessary and sufficient to limit IFN-β expression associated with tissue injury. Moreover, we also observed the addition of NEIL2 protein in *Neil2*^+/+^ and *Neil2*^−/−^ mice contributes to the host fitness correlated with diminished IFN-β expression and immunopathology.

Early studies have reported viruses as causative agents of chromosomal aberrations in cultured cells and in humans, like porcine-borne DNA virus (22), mosquito-borne RNA virus (23), influenza virus (24), and newly discovered SARS-CoV-2 (25). Notably, the DNA mismatch repair (MMR) induced by influenza virus infection is driving club cell survival (26). What we document here has broadened this field by showing the transcription-coupled (TC)-BER activated upon RSV infection protects against severe immunopathology. Reports are available implicating DNA damage was correlated mainly with ROS (4, 27). Indeed, the ROS signaling activated by RSV infection is essential for NF-κB phosphorylation (15, 28). We focused on testing the oxidative substrates of NEIL2 in the promoter because it is the regulatory region of actively transcribed DNA that is known to preferentially accumulate DNA damage (29, 30). NEIL2 is involved in the preferential repair of 5-hydroxyuracil (5-OHU) in the transcriptionally active sequences *via* the TC-BER pathway (9, 31). Our analysis of RSV-induced NEIL2 substrates in IFN-β promoter has identified that NEIL2 is not merely maintaining the genomic integrity but also has repair-independent roles in the initiation of gene expression.

Active transcription from the IFN-β promoter requires cooperative binding of *trans*-acting factors to the enhanceosome (32), beginning with the delivery of NF-κB, and followed by the binding of ATF-2/c-Jun and IRF proteins in a highly cooperative fashion (18). Our ChIP analysis showed NF-κB/RelA binding to the IFN-β promoter is an early event, proceeding IRF3/7. These results are in line with studies documenting that after IRF3/7 activation, the role of NF-κB is dispensable for IFN-β expression (33). Our results show that RSV induced a plethora of proinflammatory mediators driven by enhanced IFN-β expression in the lungs of *Neil2*^−/−^ mice. The regulation of IFN-β is involved in NEIL2 antagonizing the DNA binding of NF-κB/RelA in chromatinized DNA. To clarify NEIL2 directly interferes with the DNA binding of NF-κB/RelA, we showed that NEIL2 is co-immunoprecipitated with NF-κB/RelA. We reported previously that NEIL2 suppresses promoter occupancy of NF-κB *via* association with the Rel homology domain (10). Together with the results presented here, we provide a mechanism that in the context of the IFN-β enhancer/promoter, precisely regulated NF-κB interacting with DNA by NEIL2 sets the threshold to the transcriptional potential of positive regulatory elements to the IFN-β gene. This evidence explains the enhanced expression of IFN-β and consequently higher expression of inflammatory cytokines and chemokines in the absence of NEIL2. The antiviral responses, as shown by neutrophils infiltrated into the airway and bodyweight loss, were significantly prevented by the introduction of NEIL2 protein prior to the RSV challenge. Expression of IFN-β is essential for maintenance of the autocrine signaling, which keeps non-infected cells in an antiviral readiness state (34). Due to excessive induction of IFN-β could potentially cause damage by amplifying lung injury (5, 21), our analysis with a supplement of NEIL2 protein is of translational relevance, as the pathogenesis restriction by NEIL2 has clinical utility for antiviral therapy upon RSV infection.

Another unexpected finding of this study is that the expression of NEIL2 is decreased in RSV infected murine model but not OGG1 or MTH1. Although the mechanism of NEIL2 expression is not the focus at present, the fact of low levels of NEIL2 in RSV-infected animals warrants for the importance of NEIL2 in limiting disease severity. We have made similar observations in SARS-CoV-2 infected patients, the severity of the infection and the rate of survival are highly correlated with NEIL2 deficiency (35). In the same study, we also observed derailed cytokine and immune networks in *Neil2*^−/−^ mice that account for tissue damage. It was reported that the level of NEIL2 is significantly decreased in *Helicobacter pylori*-positive gastric cells, and lower NEIL2 expression correlated with a lower probability of survival of gastric cancer (21). Altogether, these results suggest that NEIL2 downregulation in the host is part of an antiviral response that facilitates the expression of IFN-β and consequently harmful-immune response. Treatment of RSV-infected patients is often limited to the use of bronchodilators, corticosteroids, and antiviral drugs like Ribavirin and IFNs, which are always associated with side effects (36, 37). Although these treatments are able to decrease virus replication and inflammation, the genomic damage and persistent DNA lesions caused by the virus may exacerbate the side effects and disease progression. Our study suggests the dual role of NEIL2 in DNA repair and immune modulation has clinical potential as a therapeutic strategy.

In conclusion, through molecular analysis and *Neil2*^−/−^ mice, we have presented the role of NEIL2 in controlling IFN-β levels with the potential induction of harmful immunopathology or autoimmune disorders. This detailed biochemical characterization of the NEIL2-regulated transcription in a locus-specific manner will be important to understand host defense. These results suggest a potential novel treatment modality with the administration of NEIL2 protein to halt the progression of viral-induced lung disease.

## Experimental procedures

### Cells maintenance and RSV propagation

Human small airway epithelial cells (hSAEC) (38) were cultured in Small Airway Epithelial Cell Basal Medium (C-21270, Promo Cell), supplemented with Supplement Pack (C-39170, Promo Cell). HEp-2 cells (CCL-23, ATCC) were grown in Minimum Essential Medium (MEM) (Gibco) containing 10% fetal bovine serum (Life Technologies), 100 units/ml penicillin (Gibco), and 100 μg/ml streptomycin (Gibco).

The human RSV A2 strain (VR-1544, ATCC) was propagated in HEp-2 cells at 80% confluence, washed with PBS without Ca^2+^ and Mg^2+^ and the virus was added at a multiplicity of infection of 0.1 plaque forming units (PFU)/cell diluted in serum free medium for 1 h at 37 °C. Complete medium was then added to the culture for prolonged inoculation at 37 °C. When high cytopathic effects were observed (days 4–7 p.i.), cells were scraped into the medium, cell debris was separated, and virus was purified on discontinuous sucrose gradients as described previously (39, 40). Aliquots of sucrose-purified (cytokine and lipopolysaccharide-free) RSV virion suspensions were stored at −80 °C until used. Virus titers were determined *via* plaque assay as previously described (41).

### ROS assay

CellROX Green Reagent (C10444, Thermo Fisher Scientific) was used to measure ROS levels according to the manufacturer's instruction. Briefly, hSAECs were plated on 96-well plates and pretreated with or without 10 mM of Tiron (ab146234, Abcam) and 200 mM of Trolox (53188-07-1, Sigma-Aldrich) (42) for 15 min at 37 °C, followed by RSV (MOI = 1) infection for indicated time. The cells were incubated with CellROX Green Reagent at a final concentration of 5 μM for 30 min at 37 °C, washed three times with PBS, and analyzed with a microplate reader (BioTek Synergy H1) at Ex/Em maxima 485/520 nm.

### NEIL2 protein expression and purification

Wild-type His-tagged NEIL2 proteins were purified from *E. coli* using a previously described protocol (43). In brief, vector pET22b (NovaGen) containing C-terminal His tagged-protein coding DNA sequence was transfected into *E. coli* BL21(DE3) RIPL Codon-plus cells. *E. coli* culture at log-phase (A600 = 0.6) was induced with 0.5 mM of isopropyl β-d-1-thiogalactopyranoside and incubated at 16 °C for 16 h. After sedimentation, cells were suspended in a lysis buffer (Buffer A) containing 25 mM Tris-HCl, pH 7.5, 500 mM NaCl, 10% glycerol, 1 mM ß-mercaptoethanol (ß-ME), 0.25% Tween 20, 5 mM imidazole, 2 mM phenylmethylsulphonyl fluoride, and sonicated. Cell lysates were centrifuged at 13,000 rpm at 4 °C for 15 min. The supernatant was loaded onto HisPur Cobalt Superflow Agarose (25228, Thermo Scientific previously equilibrated with Buffer A and incubated for 2 h at 4 °C. After washing with Buffer A with a gradient of increasing concentration of imidazole (10, 20, 30, 40 mM), the His-tagged proteins were eluted with an imidazole gradient (80–500 mM imidazole in buffer containing 25 mM Tris-HCl, pH-7.5, 300 mM NaCl, 10% glycerol, 1 mM ß-ME, 0.25% Tween 20). After elution, the peak protein fractions were dialyzed against Buffer C (1X PBS, pH 7.5, 1 mM dithiothreitol (DTT), and 25% glycerol) and stored at −20 °C in aliquots.

### Fragment length analysis using repair enzymes coupled comet assay

Comet assays were conducted using Fragment length analysis using repair enzymes (FLARE) Assay Kit (4040-100-FK, R&D system) in conjunction with heat-inactivated or active (Act) recombinant (r)NEIL2 digestion. To maximize the difference in the comet size, final concentration of rNEIL2 and incubation time must be optimized after several pilot experiments. Alkaline comet assay was performed on RSV-infected or mock-infected hSAECs following the manufacturer’s instructions. Tail moment was measured using Comet Assay IV v4.2 system (Perceptive Instruments). At least 50 cells were counted under each condition from three biological replicates.

### siRNA and protein transfection

ON-TARGETplus Human NEIL2 siRNA-SMARTpool (L-016345-01-0010, Dharmacon) or Non-targeting Control Pool (D-001810-10-50, Dharmacon) was transfected in hSAECs using Lipofectamine RNAiMAX reagent (13778-150, Invitrogen). The concentration of siRNA (50 nM) used to maximize *NEIL2* knockdown was optimized in pilot experiments. Cells were incubated with siRNA at least for 24 h for subsequent analysis.

Pierce protein transfection reagent kit (89850, Thermo Scientific) was used to transfect NEIL2 protein. hSAECs reached 70% to 80% confluence when carried out transfection. According to instructions, 10 μl of Pierce Reagent was used in a 6-well plate to deliver 2 μg of NEIL2 protein per well. After incubation for 3 to 4 h at 37 °C in serum-free medium, an equal volume of 20% serum-containing medium was added for incubation overnight. Cells were transfected with rNEIL2 at least for 24 h for subsequent analysis.

### Chromatin immunoprecipitation

Chromatin immunoprecipitation assays were performed as described previously (38). To avoid additional oxidative DNA damage generated during sonication, Micrococcal Nuclease digestion is used to prepare mono nucleosome. Approximately 1 × 10^7^ cells were used for fixation. Briefly, cytoplasmic protein is removed by incubating with hypotonic lysis buffer (10 mM HEPES, pH7.9, 1.5 mM MgCl_2_, 10 mM KCl) for 20 min on ice. IGEPAL CA-630 was added at a final concentration of 0.6%. After mixing thoroughly by vortex for 10 s, nuclei were collected by centrifugation at 10,000*g* for 30 s. After washing once with 1 ml of hypotonic lysis buffer, nuclei were digested with 0.5 μl of Micrococcal Nuclease (M0247, New England Biolabs) in a buffer containing 50 mM Tris-HCl and 5 mM CaCl_2_ at 37 °C for 15 min. Adding 10 μl of 0.5 M EDTA to stop digestion. Pelleting nuclei by centrifugation at 16,000*g* for 1 min at 4 °C and resuspending nuclei in lysis buffer (50 mM Tris-HCl, pH 7.4, 150 mM NaCl, 5 mM CaCl_2_, 0.25% deoxycholic acid, 1% NP-40, 1 mM EDTA, 0.1% SDS). The supernatant after lysis is used for ChIP reaction with targeted antibodies, anti-p65 (sc-372x, Santa Cruz), anti-RNA Pol II (17-620, Millipore), anti-NEIL2 (in house) (9), and IgG (sc-2025, Santa Cruz Biotechnology), overnight at 4 °C. 25 μl of protein A/G magnetic beads were added to pull down antibody–protein–DNA complex for 3 h at 4 °C. Spin briefly, wash and elute DNA for real time PCR analysis. ChIP-qPCR data were analyzed by specific Ab normalized to nonspecific (negative control) IP as previously published (10). Sequences of primers are listed in Table S1.

### Co-immunoprecipitation assay

Using nuclear compartments in co-immunoprecipitation assays was performed according to the methods described previously (38). All buffers were supplemented with complete protease inhibitor and all steps were performed on ice unless specifically defined. hSAECs were immunoprecipitated with anti-NEIL2 (10) and immunoblotted with anti-NF-κB/RelA. Immunoprecipitation was also performed with anti-NF-κB/RelA and immunoblotted with anti-NEIL2. Briefly, cytoplasmic proteins were removed, and nuclei were incubated in NLB buffer (50 mM Tris-HCl pH 7.5, 100 mM NaCl, 50 mM KCl, 3 mM MgCl_2_, 10% Glycerol, and 0.1% Tween-20). The nuclear suspension was transferred to a needle syringe (26G × 3/8) and homogenized by performing 70 strokes, followed by vortex for 30 min 250 U/ml of Benzonase Nuclease was added to digest nucleic acids for 45 min at room temperature and stopped by adding 5 mM EDTA and 5 mM EGTA. The lysate was clarified by ultracentrifugation at 20,000*g* for 15 min. The protein concentration was assessed, and 1 mg of protein was used for overnight IP with the primary antibody in rotation. Protein–antibody mixture was further incubated for 3 h with 20 μl of Protein A/G Mix Magnetic Beads, washed five times with NLB, and resuspended in 2× Laemmli buffer (1610737, Bio-Rad).

### Western blot

Protein was prepared with ice-cold 1× RIPA Lysis and Extraction Buffer (89900; Thermo Fisher Scientific) containing protease inhibitor cocktail (11836153001; Roche). After incubation for 15 min on ice, lysates were clarified by centrifugation at 16,000*g* for 10 min at 4 °C. Protein concentration was assessed using Pierce protein BCA assay kit (23225; Thermo Scientific), and 20 μg of proteins were loaded for SDS-PAGE, then transferred to polyvinylidene fluoride (PVDF) membranes. Membranes were blocked for 3 h at room temperature in TBST (0.05% Tween-20) supplemented with 5% non-fat milk. Anti-OGG1 (PA5-86046; Invitrogen), anti-MTH1 (PA5-52963, Thermo Fisher Scientific), anti-NEIL2 (PA5-78662; Invitrogen), anti-p65 (F-6) (sc-8008; Santa Cruz), anti-phospho-IRF-3 (Ser396) (4D4G) (#4947; cell signaling), anti-IRF-7 (G-8) (sc-74472, Santa Cruz) and anti-pS276 p65 (ab30623, Abcam) were used as primary antibody incubated overnight at 4 °C. β-actin (4970S; Cell Signaling Technology) and anti-Lamin A/C (636) (sc-7292, Santa Cruz) were used as internal loading control. Images were visualized by Amersham 680 imaging system.

### Electrophoretic mobility shift assays (EMSA)

EMSA assays were performed as described previously (38). Nuclear extracts (NE) were prepared using CelLytic NuCLEAR Extraction Kit (NXTRACT, Sigma) and protein concentrations were quantified by Pierce BCA Protein Assay Kit (23225, Thermo Scientific). Cy5-labeled probes were mixed with NE (1 μg) in buffer containing 10 mM HEPES (pH 7.9), 10 mM KCl, 1.5 mM MgCl_2_, 1 mM DTT, and 5% glycerol. First, proteins in reaction buffer were preincubated for 30 min on ice, and 20 nM of Cy5-labed oligo was added for another 30 min. DNA-protein complexes were resolved in 6% polyacrylamide gels (0.5 ×TBE, 5% glycerol) in 1× Tris Glycine buffer and run for 100 min at 150 V at 4 °C. Images were visualized by Amersham 680 imaging system.

### Immunofluorescent (IF) assays

Immunofluorescence protocols have been previously described (38). Briefly, cells grown on cover glasses were washed with ice-cold PBS and fixed with acetone-methanol (1:1) for 20 min at −20 °C. Cells were hydrated for 15 min in phosphate-buffered saline (PBS), and permeabilized in 0.1% (w/v) Triton-X-100 for 5 min at room temperature. cells were blocked by IgG (10 μg/ml, sc-2025, Santa Cruz) in the presence of 1% BSA for 3 h at room temperature. Primary NEIL2 antibody (1:100) (9) was incubated overnight at 4 °C. Anti-mouse Alexa 488 (A11001, Thermo Fisher Scientific) was used as secondary antibody. Images were photographed using an OLYMPUS BX53P System.

### Animals

C57BL/6 mice, free of pathogens were obtained from the Jackson Laboratories (Bar Harbor). *Neil2*^−/−^ mice on the C57BL/6J background was developed locally (9). Sixteen weeks-old mice (50% male and 50% female) were used for this study. Mice under mild anesthesia were challenged *via* the intranasal (i.n) route with purified RSV (10^6^ PFU) in 40 μL PBS per mouse, as described previously (20). Control mice obtained the same volume of PBS. In some experiments, mice were treated with 1 mg of anti-IFNAR1 antibody (clone MAR1-5A3; Leinco Technologies), or a mouse IgG1 isotype control (clone MOPC21; Bio X Cell) 1 day prior to virus infection (44). Supplement of rNEIL2 in mice was carried out as we reported previously (10, 35). Briefly, 10 μg of rNEIL2 (active or heat inactivated) was mixed with 10 μM of carrier peptide K16SP (45) and incubated for 30 min at 37 °C; then NEIL2-peptide mix was delivered to mice i.n. 72 h prior to mock or RSV infection. Animal experiments were performed according to the NIH Guide for Care and Use of Experimental Animals and approved by the University of Texas Medical Branch (UTMB) Animal Care and Use Committee (Protocol Number: 0807044D to IB, protocol no. 0606029D to TKH).

Lungs were excised and fixed in 10% buffered formalin, and paraffin embedded. Four-μm cross-sections were stained with hematoxylin and eosin. Minimum ten fields for each section were examined and analyzed to determine whether observed differences (perivasculitis, bronchiolitis, alveolitis) were statistically significant among groups. Randomly selected fields were photographed using an ECHO Revolve four Hybrid Microscope System with a built-in digital CCD color camera. Sandwich ELISA kits were used for the detection of mouse IFN-β (439407, BioLegend), IFN-γ (430804, BioLegend), IFN-λ (DY1789B, R&D Systems) levels.

To evaluate cellular recruitment into the airway lumen, bronchoalveolar lavage (BAL) was performed twice with 0.75 ml of sterile PBS (pH 7.3). Cells were resuspended in red blood cell lysis buffer (R7757-100ML, Millipore-Sigma) for 5 min on ice and centrifuged. After sedimentation (800*g* for 5 min at 4 °C), the resulting supernatants were stored at −80 °C for further analysis. Total cell counts in the BALF were determined from an aliquot of the cell suspension using a hemocytometer. Cytospin slides were prepared using a Shandon CytospinR 4 Cytocentrifuge (Thermo Scientific) and stained with Wright–Giemsa stain kit (9990710, Thermo Scientific) using a Hematec Slide Stainer. Differential cell counts were performed in a blinded fashion by two independent researchers counting 1000 cells from each animal.

### Bio-Plex Chemokine analysis

For the detection of multiple chemokines in BALF, the Bio-Plex Pro Mouse Chemokine Panel 31-Plex (12009159, Bio-Rad) was used on a Luminex-xMAP/Bio-Plex 200 System with Bio-Plex Manager 6.2 software (Bio-Rad). A cytometric magnetic bead-based assay was used to measure cytokine levels, according to the instructions.

### Quantitative real-time PCR and gene expression profiling

Total RNAs were extracted using RNeasy Mini kit (Qiagen) as recommended by the manufacturer. Samples were treated with DNase I and subjected to the following steps. After qualification, 1 μg of total RNAs were reverse transcribed to cDNAs using the iScript reverse transcription supermix (Bio-Rad). qRT-PCR was completed using Bio-Rad CFX96 Detection System. Changes in mRNA levels were determined using the 2^−ΔΔCt^ method (46). Sequences of primers are listed in Table S2.

After various time RSV-infection, animals were euthanized, lungs were removed, and homogenized. cDNA from individual mouse was subjected to the RT^2^ Profiler PCR Array (PAMM-011ZD, Mouse Inflammatory Cytokines & Receptors, Qiagen). Hierarchical clusters for 79 expressing genes were constructed with Morpheus (https://software.broadinstitute.org/morpheus/), and volcano plots were generated in MS Excel using mean fold change and *p* values calculated from three biological replicates.

### Statistical analysis

Results were analyzed for significant differences between the two groups using an unpaired Student’s *t* test. A two-way ANOVA is used to determine the difference between the means of three or more independent groups that have been split on two variables. Differences were considered significant at *p* <0.05.

## Data availability

The data that support the findings of this study are available from the corresponding author upon request.

## Supporting information

This article contains supporting information.

## Conflict of interest

The authors declare that they have no conflicts of interest with the contents of this article.
