# Supplementary material for: Nei-like DNA glycosylase 2 selectively antagonizes interferon-β expression upon respiratory syncytial virus infection
Source: J Biol Chem. 2023 Jul 8;299(8):105028. doi: 10.1016/j.jbc.2023.105028 (PMC10403741; doi:10.1016/j.jbc.2023.105028)
Supplement: Supporting Figures S1–S8 and Tables S1 and S2 [file mmc1.docx]

**Supporting information**

**Nei-like DNA glycosylase 2 (NEIL2) selectively antagonizes interferon-β expression upon respiratory syncytial virus infection**

Lang Pan^1, #^, Yaoyao Xue^1, #^, Ke Wang^1^, Xu Zheng^1^, Azharul Islam^2^, Nisha Tapryal^2^, Anirban Chakraborty^2^, Attila Bacsi^3^, Xueqing Ba^4^, Tapas K. Hazra^2^, Istvan Boldogh^1,^ *

From the ^1^Department of Microbiology and Immunology, ^2^Department of Internal Medicine, University of Texas Medical Branch at Galveston, Galveston, TX 77555, USA; ^3^Department of Immunology, Faculty of Medicine, University of Debrecen, Hungary; Debrecen H-4012, Hungary; ^4^Key Laboratory of Molecular Epigenetics of Ministry of Education, School of Life Science, Northeast Normal University, Changchun, Jilin, China.

^#^Contribute equally

*Corresponding author: Istvan Boldogh, Email: [sboldogh@utmb.edu](mailto:sboldogh@utmb.edu)

**Running title:**

NEIL2 antagonizes interferon-β expression upon RSV infection.

**Key words:** base excision repair, DNA damage, negative-strand RNA virus, NF-κB, transcription regulation, inflammation, innate immunity, antiviral response

**List of Supporting Material:**

1. Figure S1-S8

2. Table S1

3. Table S2


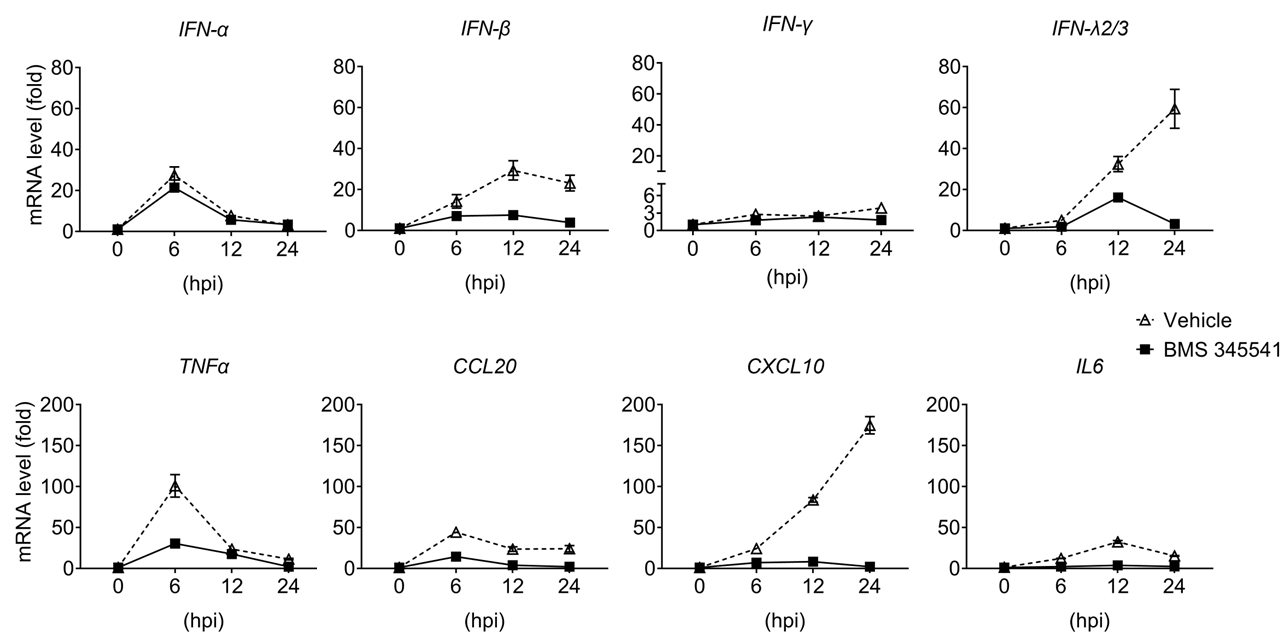


**Figure S1**: NF-κB mediates RSV-induced gene expression. hSAECs were pretreated with vehicle or IKK inhibitor BMS 345541 (5 µM) for 1 hour and infected by RSV (1 MOI). At indicated time post infection, total RNA was extracted, and mRNA level was measured by qRT-PCR. Data is presented as mean ± SD with three technical replicates.


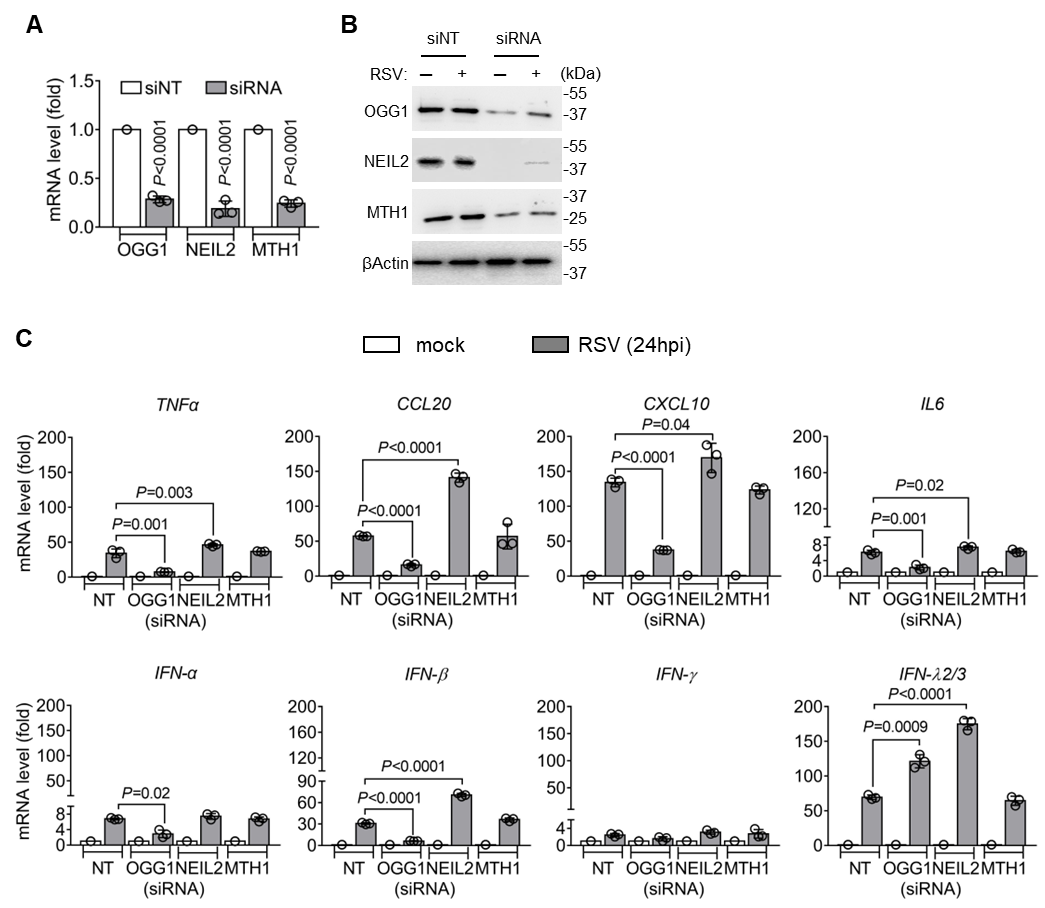


**Figure S2**: NEIL2 suppresses RSV-induced gene activation.

*A*, hSAECs were transfected with non-targeted (NT) and OGG1, NEIL2, MTH1 targeted siRNA for 24 hours, followed by RSV infection (1 MOI) for 24 hours. Total RNA was extracted, and mRNA levels were measured by qRT-PCR. *B*, Whole cell lysates from *A* were prepared and subjected to western blot analysis with antibodies against OGG1, NEIL2 and MTH1. *C*, Total RNA was extracted as in *A*, and mRNA levels of IFNs and inflammatory genes were measured by qRT-PCR. Data is presented as mean ± SD with three technical replicates. *p* values were calculated by unpaired Student’s *t*-tests (two-tailed).


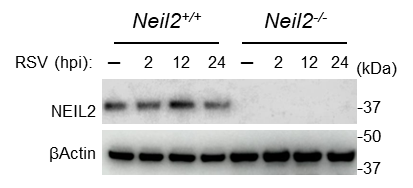


**Figure S3**: The protein level of NEIL2 in the lungs of *Neil2*^+/+^ and *Neil2*^−/−^ mice as shown by western blot. In each group, lung lysates from 6 mice were pooled from three biological replicates.


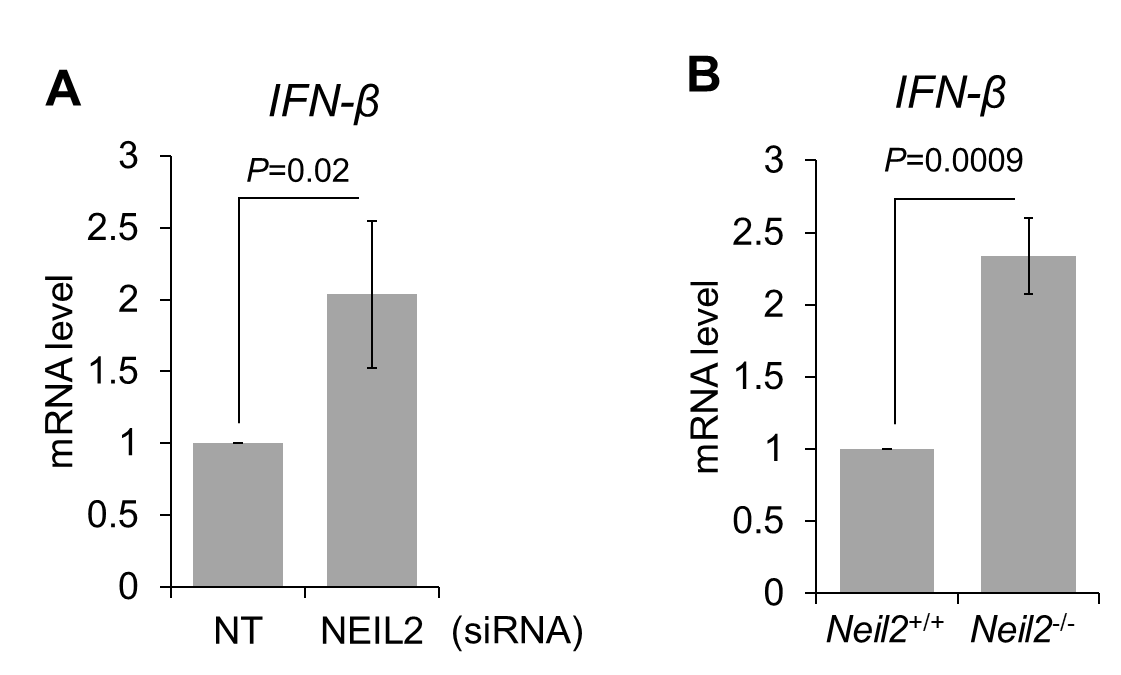


**Figure S4**: The expression level of IFN-β at steady state is higher in the absence of NEIL2.

*A*, Total RNA was extracted from mock-infected hSAECs. *B*, Total RNA was extracted from mock-infected *Neil2*^+/+^ and *Neil2*^-/-^ mice. The mRNA level of IFN-β was measured by qRT-PCR. Data is presented as mean ± SD with three technical replicates. *p* values were calculated by unpaired Student’s *t*-tests (two-tailed).


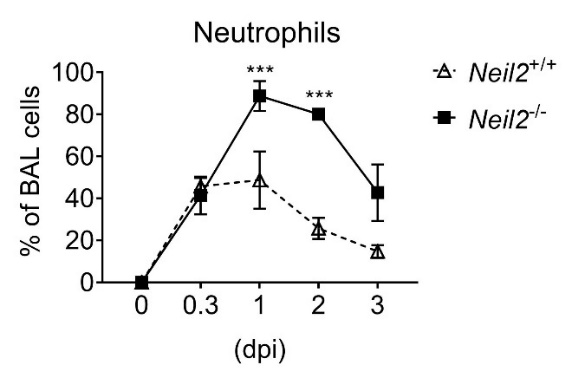


**Figure S5**: Percentage of neutrophils in the BALF at indicated days post infection (dpi). Data shown is pooled data from 3 biological replicates with 5 mice per group in each experiment. *p* values were calculated by two-way ANOVA. ****p* < 0.001.

**
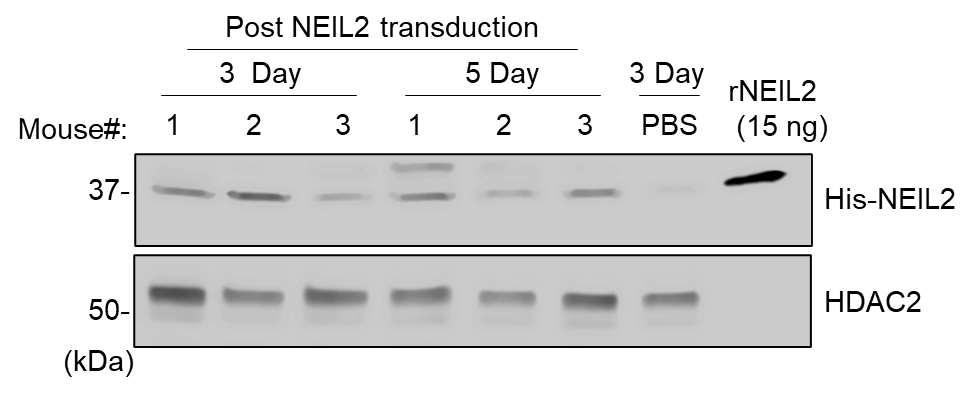
**

**Figure S6**: rNEIL2 levels in the mice lungs after transduction. After rNEIL2 was delivered to mice via intranasal (i.n) route for indicated time, lungs (n=3) were homogenized and subjected to western blot with anti-His antibody. His-NEIL2 was used as positive control and HDAC2 was used as internal loading control. Representative image is shown from three biological replicates.


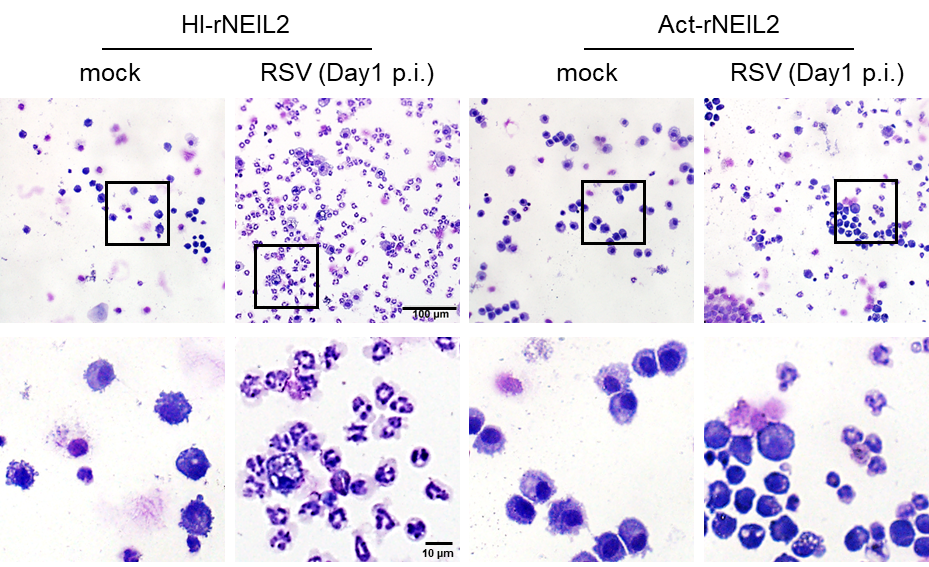


**Figure S7**: Representative images of infiltrate cells in BALF from *Neil2*^−/−^ mice supplemented heat-inactivated (HI) and activate (Act) rNEIL2. The scale bar represents 100 µm in lower magnification, and 10 µm with zoom in.


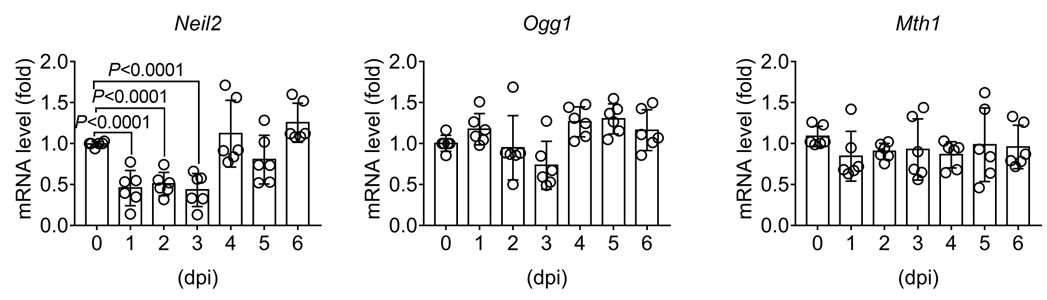


**Figure S8**: Decreased NEIL2 expression after RSV infection. C57BL/6 mice were infected with RSV (10^6^ PFU) for indicated time, and total RNA was isolated from lungs. mRNA levels of NEIL2, OGG1 and MTH1 were measured by qRT-PCR at indicated days p.i. Data is expressed as mean ± SD (n=6 mice from three biological replicates). *p* values were calculated by unpaired Student’s *t*-tests (two-tailed).

**Table S1**. Sequences of primers used in ChIP coupled real time-PCR.

| Target | Forward (5’-3’) | Reverse (5’-3’) |
| --- | --- | --- |
| IFN-α (-184 / -4) | AAGGCTCTGGGGTAAAAGA | GACCTTGCTTTGTGCCTAGC |
| IFNβ (-150 / +5) | tcataagataggagcttaaa | GAATGTCCTTTCTCCATGG |
| IFNγ (-142 / +54) | AATGCCACAAAACCTTAGTTATTAA | ACTTAACTGATCTTTCTCTTCTAAT |
| IFNλ (-204 / -17) | CTGTAGGTTACCCCTGAGTC | CTGTAGGTTACCCCTGAGTC |
| Ifnα (-236 / -64) | ttgaacccacattccccagg | ttgaacccacattccccagg |
| Ifnβ (-281 / -34) | agggctgtctcctttctgtt | gatggtcctttctgcctcag |
| Ifnγ (-282 / -119) | tcatcgtcagagagcccaag | gaaggctcctcgggattacg |
| Ifnλ2/3 (-482 / -288) | gtagcctcctgacgaacctt | ttaacctggtctgatgggca |

**Table S2**. qRT-PCR primer used in this study for mRNA analysis.

| Target | Forward (5’-3’) | Reverse (5’-3’) |
| --- | --- | --- |
| IFNA1 | CCCATTTCAACCAGTCTAGCAG | TGTGGGTTTGAGGCAGATC |
| IFNB1 | TGCTCTGGCACAACAGGTAG | CAGGAGAGCAATTTGGAGGA |
| IFNG | CCAACGCAAAGCAATACAAGA | TTTTCGCTTCCCTGTTTTAGC |
| IFNL2/3 | AGTGCTGACCGTGACTGGA | GCGACTCTTCTAAGGCATCTT |
| NEIL2 | CATCGAGGACAAGCCTTAGAAGC | CACTCAGGACTGAACCGAGAGA |
| OGG1 | CATATGAGGAGGCCCACAAG | CAGAAGATAAGAGGACGCAGAAG |
| MTH1 | GTCTTCTGCACAGACAGCATCC | CTGAAGCAGGAGTGGAAACCAG |
| TNFα | TGCACTTTGGAGTGATCGG | TCAGCTTGAGGGTTTGCTAC |
| CCL20 | CCATGTGCTGTACCAAGAGT | TTAGGATGAAGAATACGGTCTGTG |
| CXCL10 | GACATATTCTGAGCCTACAGCA | CAGTTCTAGAGAGAGGTACTCCT |
| IL6 | GCAGATGAGTACAAAAGTCCTGA | TTCTGTGCCTGCAGCTTC |
| GAPDH | ACATCGCTC AGACACCATG | TGTAGTTGAGGTCAATGAAGGG |
| Ogg1 | ATTGCACTGTGTACCGAGGA | GGGTCTTGTCTCAGCAGTCT |
| Mth1 (Nudt1) | CTCGGAGCACTAGTTTTCGC | GTCTCTCCTTCCTGCACCTT |
| Neil2 | AGTAAGAAGCTCCACCCTGC | GCCTCTTTCTGCCACATTCC |
| Ifna1 | ATGGCTAGGCTCTGTGCTTTCC | TCCTCACAGCCAGCAGGGAGT |
| Ifnb1 | CAGCTCCAAGAAAGGACGAAC | GGCAGTGTAACTCTTCTGCAT |
| Ifng | GCTTTGCAGCTCTTCCTCA | TTTCTTCCACATCTATGCCACT |
| Ifnl2/3 | TCCTCCTGCTGTTGCCTCTG | GGTGGGAACTGCACCTCA |
| Gapdh | AATGGTGAAGGTCGGTGTG | GTGGAGTCATACTGGAACATGTAG |
| Tnfα | AGACCCTCACACTCAGATCA | TCTTTGAGATCCATGCCGTTG- |
| Cxcl10 | ATTTTCTGCCTCATCCTGCT | TGATTTCAAGCTTCCCTATGGC |
| Ccl20 | CCAGCACTGAGTACATCAACT | GTATGTACGAGAGGCAACAGTC |
| Il6 | AGCCAGAGTCCTTCAGAGA | TCCTTAGCCACTCCTTCTGT |
